# Supplementary material for: Long-term risk of psychiatric disorder and psychotropic prescription after SARS-CoV-2 infection among UK general population
Source: Nat Hum Behav. 2024 Mar 21;8(6):1076–87. doi: 10.1038/s41562-024-01853-4 (PMC11199144; doi:10.1038/s41562-024-01853-4)
Supplement: Supplementary file 1 — Supplementary Figs. 1–6 and Tables 1–9. [file 41562_2024_1853_MOESM1_ESM.pdf]

nature human behaviour

Article

https://doi.org/10.1038/s41562-024-01853-4

# Long-term risk of psychiatric disorder and psychotropic prescription after SARS-CoV-2 infection among UK general population

In the format provided by the authors and unedited

**Long-term risk of psychiatric disorder and psychotropic prescription after SARS-CoV-2 infection among UK general population**


| Table of Contents | Page |
|---|---|
| **Supplementary Figures** | |
| Supplementary Fig. 1. Distribution of SARS-CoV-2 infections in the UK Biobank over the study period | **1** |
| Supplementary Fig. 2. Standardized mean differences between the SARS-CoV-2 infection group and the contemporary control group before and after weighting by mental health outcome | **2** |
| Supplementary Fig. 3. Risks of first or recurrent psychiatric diagnoses and psychotropic prescriptions after SARS-CoV-2 infection compared with the contemporary control group | **3** |
| Supplementary Fig. 4. Risks of first psychiatric diagnoses and psychotropic prescriptions after SARS-CoV-2 infection compared with the historical control group | **4** |
| Supplementary Fig. 5. Risks of first or recurrent psychiatric diagnoses and psychotropic prescriptions after SARS-CoV-2 infection compared with the historical control group | **5** |
| Supplementary Fig. 6. Risks of composite mental health outcomes after SARS-CoV-2 infection compared with the historical control group | **6** |
| **Supplementary Tables** | |
| Supplementary Table 1. Definition of mental health related outcomes | **7** |
| Supplementary Table 2. Demographic and medical characteristics of the SARS-CoV-2 infection, contemporary control, and historical control groups before weighting | **8** |
| Supplementary Table 3. Demographic and medical characteristics of the SARS-CoV-2 infection, contemporary control, and historical control groups (without history of mental health outcome two years before the start of follow-up) before weighting | **10** |
| Supplementary Table 4. Demographic and medical characteristics of the SARS-CoV-2 infection, contemporary control, and historical control groups (without history of mental health outcome two years before the start of follow-up) after weighting | **12** |
| Supplementary Table 5. Number of individuals with a history of mental health outcome in the past two years before the start of follow up | **14** |
| Supplementary Table 6. Risks of composite incident mental health outcomes after SARS-CoV-2 infection compared with the test-negative control group | **15** |
| Supplementary Table 7. Positive and negative outcome controls | **16** |
| Supplementary Table 8. Sensitivity analyses of the risks of composite incident mental health outcomes | **17** |
| Supplementary Table 9. Methodological pitfalls in long-COVID study using electronic health records compared with community-based cohort study | **18** |

**Supplementary Fig. 1 Distribution of SARS-CoV-2 infections in the UK Biobank over the study period**

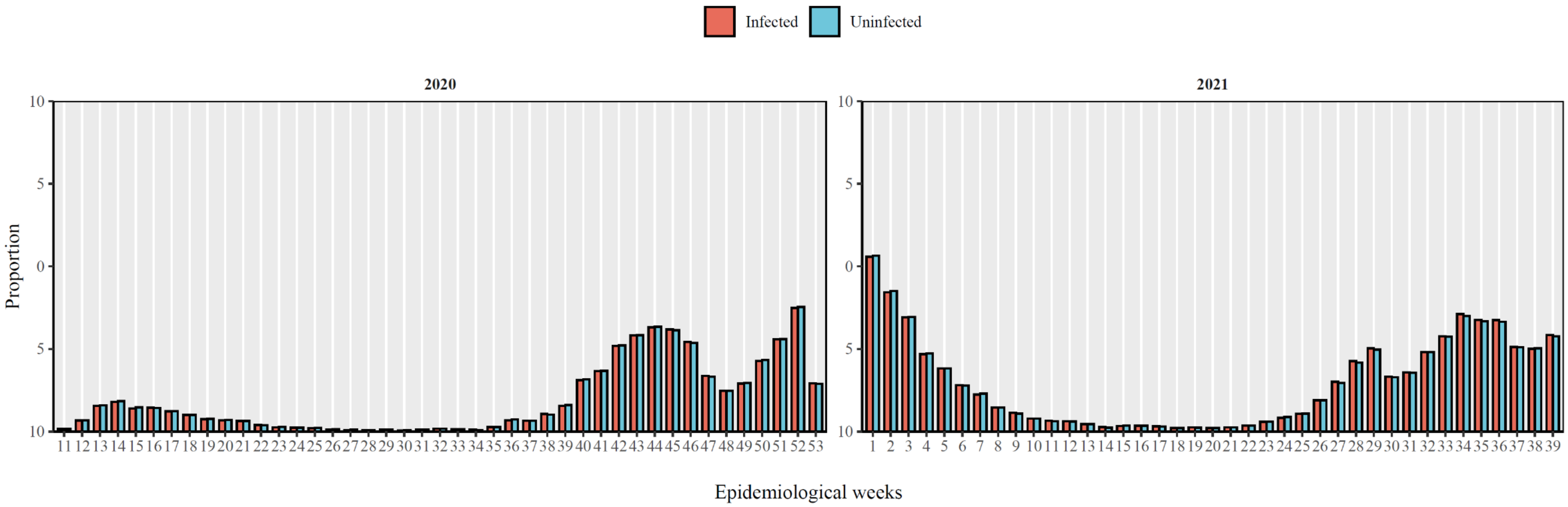

**Supplementary Fig. 2 Standardized mean differences between the SARS-CoV-2 infection group and the contemporary control group before and after weighting by mental health outcome**

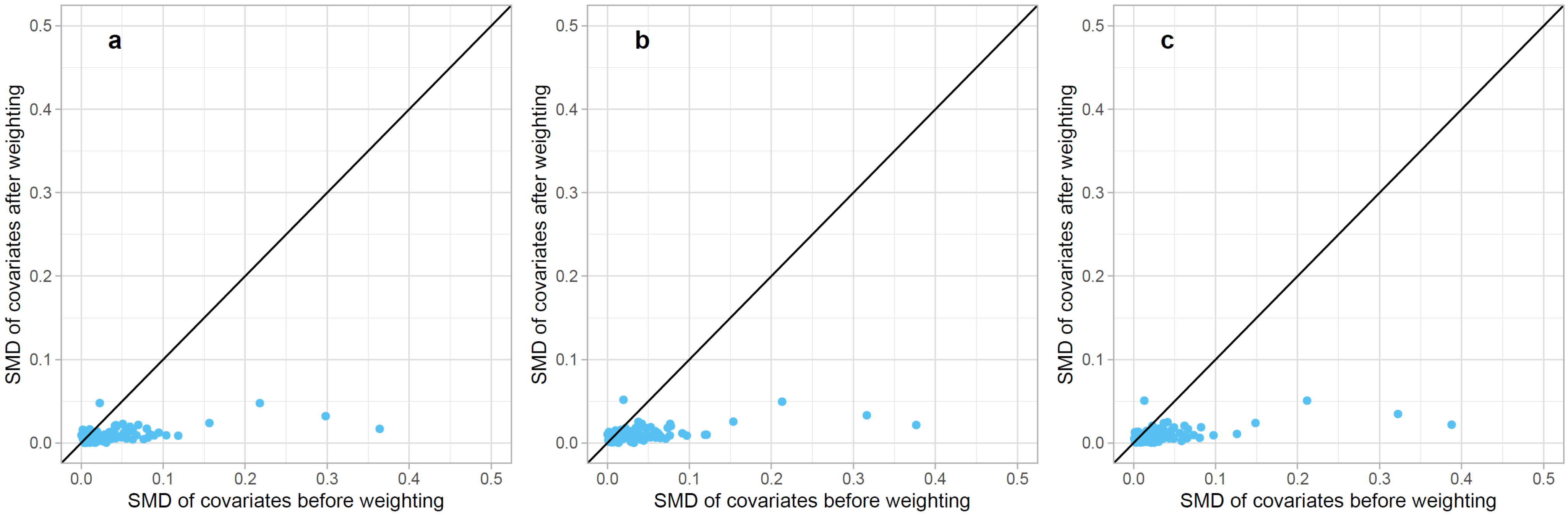


**a, any psychiatric diagnosis; b, any psychotropic medication; c, any mental health outcome.**

**Supplementary Fig. 3 Risks of first or recurrent psychiatric diagnoses and prescriptions for psychotropic medications after SARS-CoV-2 infection compared with the contemporary control group**

**A**

**Hazard ratio**

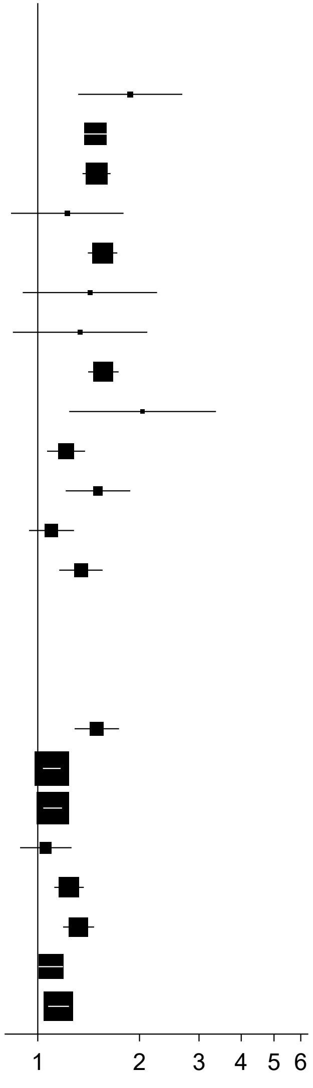


| | No. of events | HR (95% CI) |
|---|---|---|
| **Psychiatric diagnoses** | | |
| Psychotic disorders | 347 | 1.88 (1.32-2.68) |
| Mood disorders | 5699 | 1.48 (1.35-1.62) |
| Depressive episode | 5377 | 1.49 (1.36-1.64) |
| Mania/Bipolar affective disorder | 377 | 1.22 (0.83-1.79) |
| Anxiety disorders | 5096 | 1.56 (1.41-1.72) |
| Phobic anxiety disorder | 294 | 1.43 (0.90-2.25) |
| Panic disorder | 284 | 1.33 (0.84-2.11) |
| Generalized anxiety disorder | 4698 | 1.56 (1.41-1.73) |
| PTSD | 131 | 2.04 (1.24-3.37) |
| Substance use disorders | 4054 | 1.21 (1.07-1.38) |
| Alcohol use disorder | 1154 | 1.51 (1.21-1.88) |
| Tobacco use disorder | 3129 | 1.10 (0.94-1.28) |
| Sleep disorders | 2230 | 1.34 (1.16-1.56) |
| **Psychotropic medications** | | |
| Antipsychotics | 2237 | 1.50 (1.29-1.74) |
| Antidepressants | 17549 | 1.10 (1.04-1.17) |
| SSRI | 15564 | 1.11 (1.04-1.18) |
| SNRI | 2096 | 1.06 (0.89-1.26) |
| Other antidepressants | 6270 | 1.24 (1.12-1.37) |
| Benzodiazepines | 5648 | 1.32 (1.19-1.47) |
| Mood stabilizers | 10238 | 1.09 (1.01-1.19) |
| Opioids | 13942 | 1.15 (1.07-1.24) |

**B**

**Absolute risk difference**

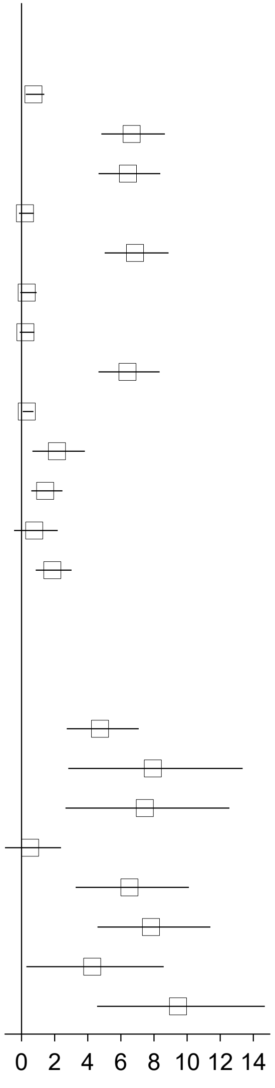


| | Risk difference per 1000 person-years (95% CI) |
|---|---|
| **Psychiatric diagnoses** | |
| Psychotic disorders | 0.72 (0.26 to 1.37) |
| Mood disorders | 6.65 (4.82 to 8.66) |
| Depressive episode | 6.43 (4.65 to 8.38) |
| Mania/Bipolar affective disorder | 0.21 (-0.15 to 0.74) |
| Anxiety disorders | 6.85 (5.02 to 8.88) |
| Phobic anxiety disorder | 0.31 (-0.07 to 0.91) |
| Panic disorder | 0.23 (-0.11 to 0.77) |
| Generalized anxiety disorder | 6.40 (4.65 to 8.34) |
| PTSD | 0.32 (0.07 to 0.72) |
| Substance use disorders | 2.14 (0.66 to 3.82) |
| Alcohol use disorder | 1.43 (0.59 to 2.47) |
| Tobacco use disorder | 0.77 (-0.45 to 2.18) |
| Sleep disorders | 1.86 (0.86 to 3.02) |
| **Psychotropic medications** | |
| Antipsychotics | 4.74 (2.73 to 7.08) |
| Antidepressants | 7.93 (2.82 to 13.35) |
| SSRI | 7.45 (2.65 to 12.55) |
| SNRI | 0.52 (-1.05 to 2.38) |
| Other antidepressants | 6.52 (3.27 to 10.11) |
| Benzodiazepines | 7.82 (4.59 to 11.41) |
| Mood stabilizers | 4.27 (0.29 to 8.59) |
| Opioids | 9.45 (4.56 to 14.69) |

Mental health outcomes were ascertained after the SARS-CoV-2 infection until the end of follow-up. Hazard ratios were adjusted for predefined and data-driven covariates. Squares represent estimates of HRs or risk difference, and error bars represent the corresponding 95% CIs. SSRI=selective serotonin reuptake inhibitor; SNRI =serotonin-noradrenaline reuptake inhibitor.

**Supplementary Fig. 4 Risks of first psychiatric diagnoses and prescriptions for psychotropic medications after SARS-CoV-2 infection compared with the historical control group**

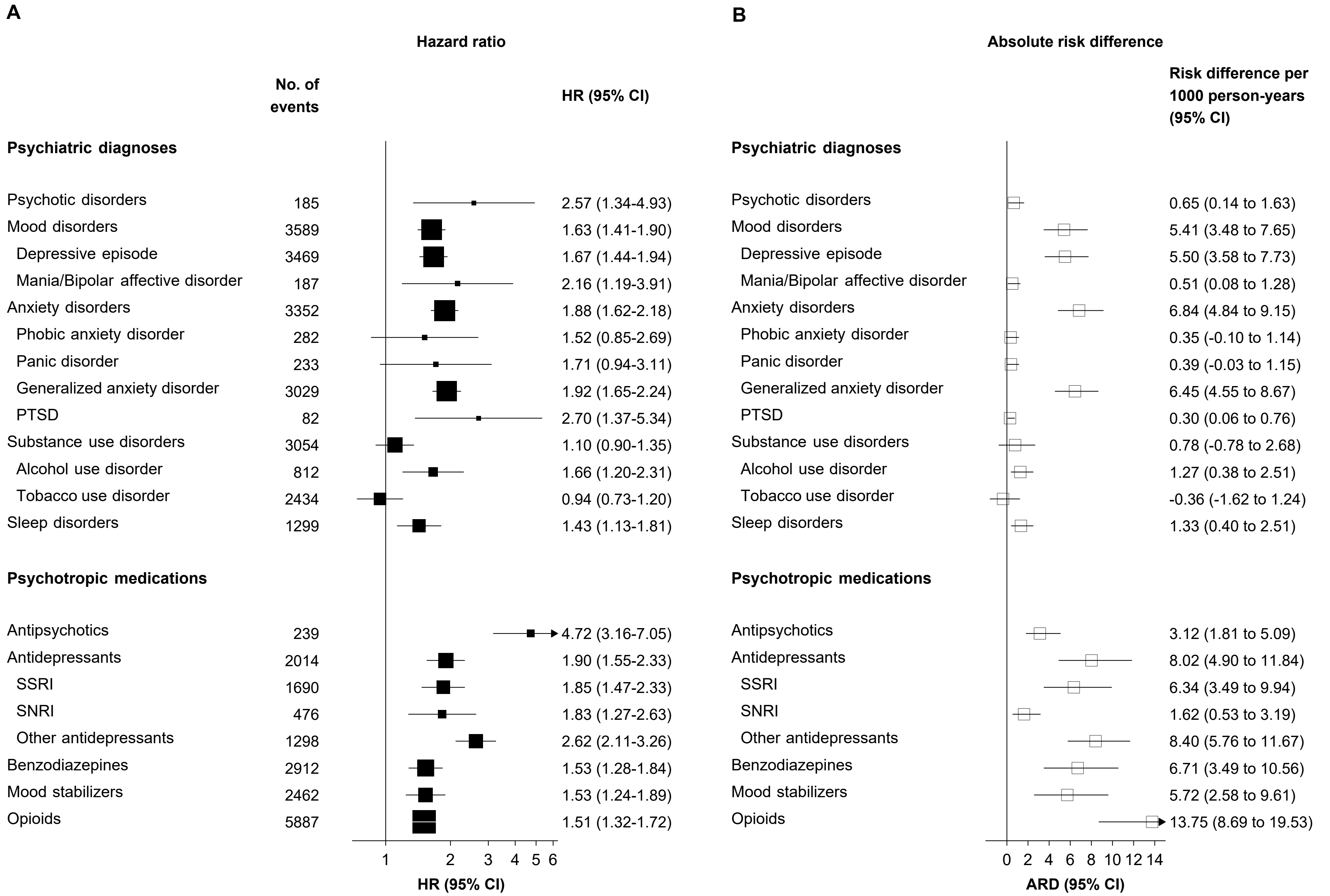


Mental health outcomes were ascertained after the SARS-CoV-2 infection until the end of follow-up. Hazard ratios were adjusted for predefined and data-driven covariates. Squares represent estimates of HRs or risk difference, and error bars represent the corresponding 95% CIs. SSRI=selective serotonin reuptake inhibitor; SNRI =serotonin-noradrenaline reuptake inhibitor.

**Supplementary Fig. 5 Risks of first or recurrent psychiatric diagnoses and prescriptions for psychotropic medications after SARS-CoV-2 infection compared with the historical control group**

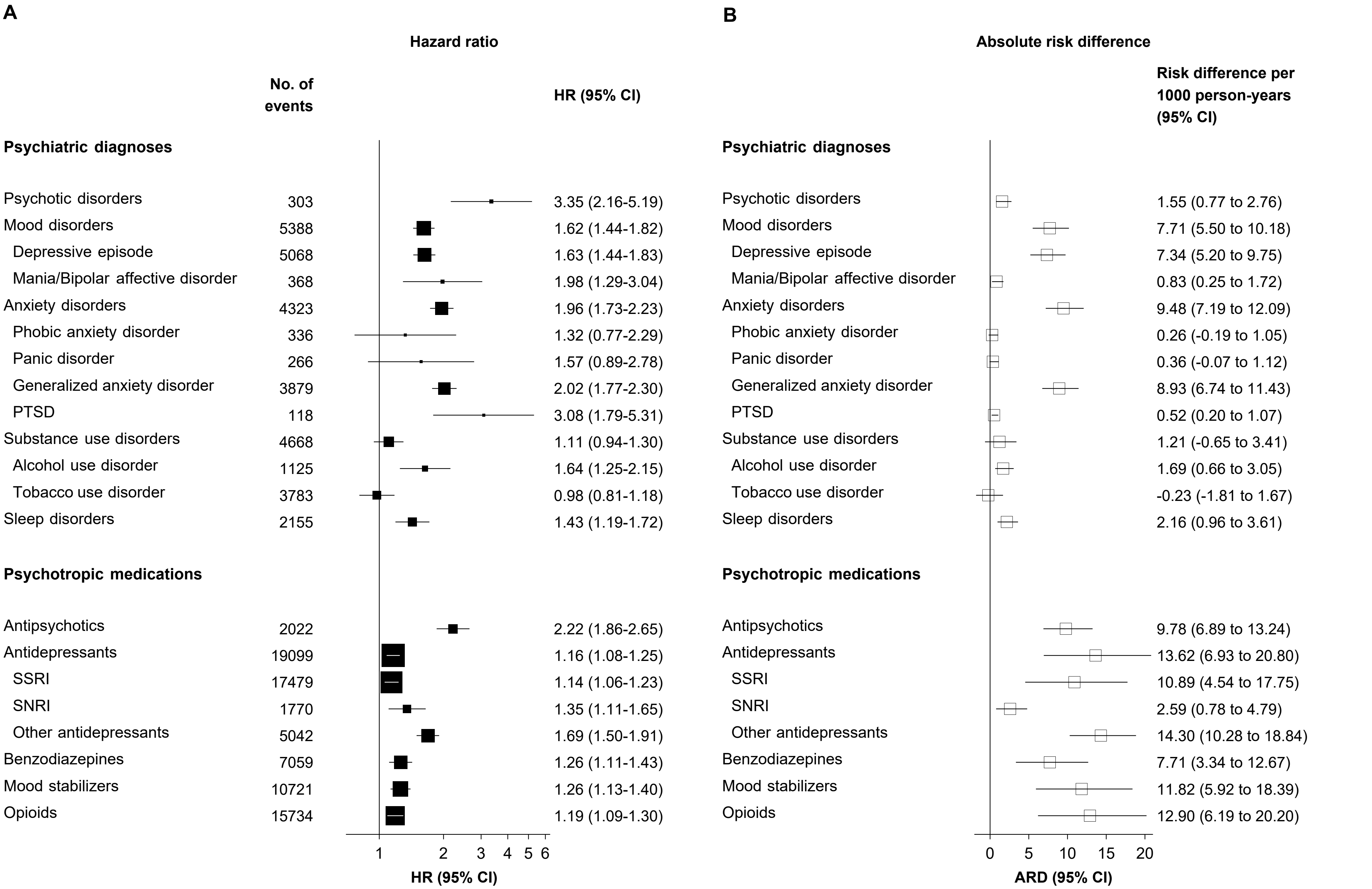


Mental health outcomes were ascertained after the SARS-CoV-2 infection until the end of follow-up. Hazard ratios were adjusted for predefined and data-driven covariates. Squares represent estimates of HRs or risk difference, and error bars represent the corresponding 95% CIs. SSRI=selective serotonin reuptake inhibitor; SNRI =serotonin-noradrenaline reuptake inhibitor.

**Supplementary Fig. 6 Risks of composite mental health outcomes after SARS-CoV-2 infection compared with the historical control group A**

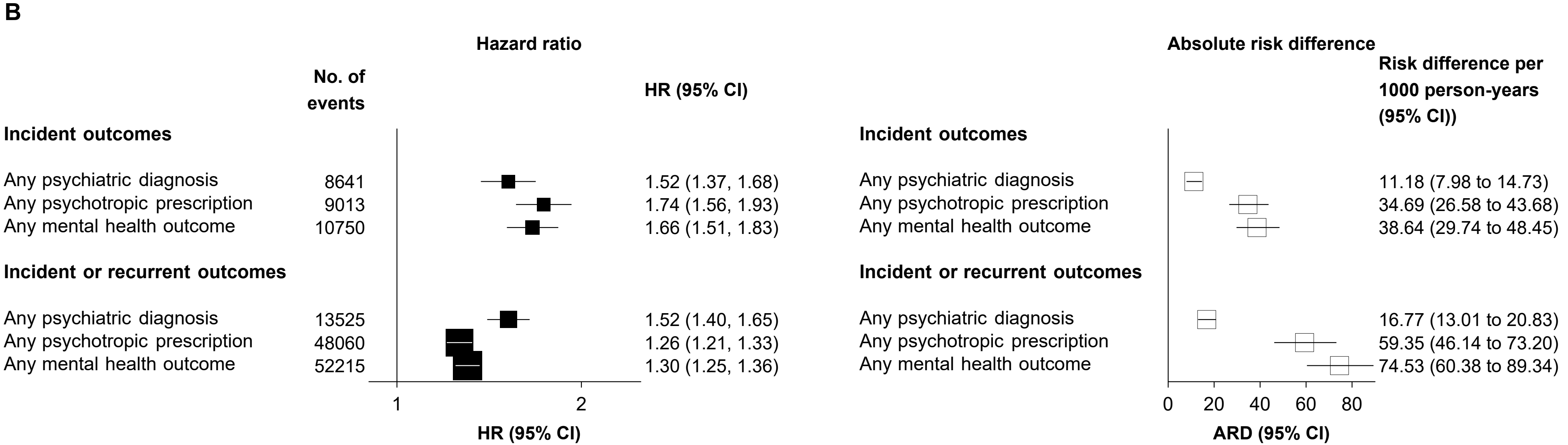


Mental health outcomes were ascertained after the SARS-CoV-2 infection until the end of follow-up. Hazard ratios were adjusted for predefined and data-driven covariates. Squares represent estimates of HRs or risk difference, and error bars represent the corresponding 95% CIs.

**Supplementary Table 1. Definition of mental health related outcomes**

| Outcome | Type of definition | Definition |
|---|---|---|
| **Psychiatric diagnoses** | | |
| Psychotic disorders | ICD-10 | F20-F29 |
| Mood disorders | ICD-10 | F30-F39 |
| Mania/Bipolar affective disorder | ICD-10 | F30-F31 |
| Depressive episode | ICD-10 | F32 |
| Anxiety disorders | ICD-10 | F40-F48 |
| Phobic anxiety disorders | ICD-10 | F40 |
| Panic disorder | ICD-10 | F41.0 |
| Generalized anxiety disorder | ICD-10 | F41.1 |
| Posttraumatic stress disorder | ICD-10 | F43.1 |
| Substance use disorders | ICD-10 | F10-F19 |
| Alcohol use disorder | ICD-10 | F10 |
| Tobacco use disorder | ICD-10 | F17 |
| Sleep disorders | ICD-10 | F51, G47 |
| **Prescriptions for psychotropic medications** | | |
| Antipsychotics | Medication | Amisulpride, Aripiprazole, Clozapine, Olanzapine, Paliperidone, Quetiapine, Risperidone |
| Antidepressant | Medication | Any SSRI, SNRI, or other antidepressant medications |
| SSRI | Medication | Citalopram, Escitalopram, Dapoxetine, Escitalopram, Fluoxetine, Fluvoxamine, Paroxetine, Sertraline, Vortioxetine, Trazodone |
| SNRI | Medication | Venlafaxine, Duloxetine, Milnacipran |
| Other antidepressant drugs | Medication | Flupentixol, Doxepin, Bupropion, Amoxapine, Maprotiline, Mianserin, Mirtazapine |
| Benzodiazepines | Medication | Diazepam, Nitrazepam, Lorazepam, Flurazepam, Temazepam, Flunitrazepam, Alprazolam, Clobazam, Clonazepam, Chlordiazepoxide, Lormetazepam, Oxazepam, Midazolam |
| Mood stabilizers | Medication | Lithium, Carbamazepine, Oxcarbazepine, Lamotrigine, Valproate, Valproic acid, Sodium valproate, Topiramate, Gabapentin |
| Opioids | | |

| | | |
|---|---|---|
| Opioid prescription | Medication | Codeine, Hydromorphone, Morphine, Opium, Oxycodone, Alfentanil, Dihydrocodeine, Diphenoxylate, Fentanyl, Remifentanil, Pentazocine, Tramadol, Nalbuphine |
| Naloxone or naltrexone | Medication | Naloxone or naltrexone |
| Methadone | Medication | Methadone |
| Buprenorphine | Medication | Buprenorphine |
| **Composite outcomes** | | |
| Any psychiatric diagnosis | Composite | Any ICD-10 code listed |
| Any prescriptions for psychotropic medications | Composite | Any psychotic medication listed |
| Any mental health related conditions | Composite | Any ICD-10 code or psychotic medication listed |

**Supplementary Table 2. Demographic and medical characteristics of the SARS-CoV-2 infection, contemporary control, and historical control groups before weighting**

| Characteristics | SARS-CoV-2 infection (n=26101) | Contemporary control (n=380337) | Historical control (n=390621) | ASMD between infection and contemporary control* | ASMD between infection and historical control* |
|---|---|---|---|---|---|
| Age, mean (sd) | 66.0 (8.5) | 68.8 (8.1) | 66.0 (8.1) | 0.34 | 0.01 |
| Sex, male (%) | 12294 (47.1) | 169325 (44.5) | 175251 (44.9) | 0.05 | 0.05 |
| Ethnicity, White (%) | 22081 (84.6) | 356477 (93.7) | 366312 (93.8) | 0.30 | 0.30 |
| Index of Multiple Deprivation, mean (sd) | 20.5 (14.9) | 17.3 (13.7) | 17.4 (13.8) | 0.22 | 0.22 |
| Body Mass Index, mean (sd) | 28.1 (5.0) | 27.3 (4.7) | 27.3 (4.7) | 0.17 | 0.16 |
| Current smoker (%) | 2845 (10.9) | 36696 (9.6) | 37312 (9.7) | 0.01 | 0.23 |
| Current drinker (%) | 23700 (90.8) | 349710 (91.9) | 352987 (91.9) | 0.11 | 0.05 |
| Physical activity, high level (%)[#] | 8535 (32.7) | 124598 (32.8) | 125638 (32.7) | 0.05 | 0.06 |
| Vaccination status, fully-vaccinated (%) | 9710 (37.2) | 148748 (39.1) | NA | 0.04 | NA |
| Medications (%)[†] | | | | | |
| Lipid lowering drugs | 8666 (33.2) | 135500 (35.6) | 119856 (30.7) | 0.05 | 0.06 |
| RAS inhibitors | 6082 (23.3) | 90955 (23.9) | 85751 (22.0) | 0.02 | 0.03 |
| Other anti-hypertensives | 2871 (11) | 41110 (10.8) | 40661 (10.4) | 0.01 | 0.02 |
| Anticoagulants | 1122 (4.3) | 16313 (4.3) | 12189 (3.1) | 0.01 | 0.06 |
| Antiplatelet drugs | 3028 (11.6) | 42667 (11.2) | 41524 (10.6) | 0.01 | 0.03 |
| Proton pump inhibitors | 8457 (32.4) | 110808 (29.1) | 101608 (26.0) | 0.07 | 0.15 |
| Diabetes medicines | 2219 (8.5) | 25562 (6.7) | 23657 (6.1) | 0.07 | 0.09 |
| Systemic glucocorticoids | 1749 (6.7) | 19910 (5.2) | 24043 (6.2) | 0.06 | 0.02 |
| Immunosuppressants | 339 (1.3) | 4629 (1.2) | 4495 (1.2) | 0.01 | 0.02 |
| Antineoplastic agents | 26 (0.1) | 411 (0.1) | 350 (0.1) | 0.01 | 0.02 |
| Coexisting conditions (%)[†] | | | | | |
| Acquired immunodeficiency syndrome | 27 (0.1) | 394 (0.1) | 384 (0.1) | 0.01 | 0.01 |
| Cancer | 2401 (9.2) | 40844 (10.7) | 36029 (9.3) | 0.05 | 0.04 |
| Cerebrovascular disease | 626 (2.4) | 8817 (2.3) | 7061 (1.8) | 0.01 | 0.04 |
| Chronic obstructive pulmonary disease | 4803 (18.4) | 62960 (16.6) | 59503 (15.2) | 0.05 | 0.09 |
| Chronic kidney disease | 1383 (5.3) | 20092 (5.3) | 15827 (4.1) | 0.01 | 0.06 |

| | | | | | |
|---|---|---|---|---|---|
| Congestive heart failure | 506 (1.9) | 5681 (1.5) | 3692 (0.9) | 0.03 | 0.08 |
| Dementia | 496 (1.9) | 3026 (0.8) | 1494 (0.4) | 0.09 | 0.14 |
| Diabetes (uncomplicated) | 2949 (11.3) | 36119 (9.5) | 31121 (8.0) | 0.06 | 0.11 |
| Diabetes (end-organ damage) | 966 (3.7) | 11451 (3.0) | 9454 (2.4) | 0.04 | 0.07 |
| Hemiplegia | 36 (0.1) | 416 (0.1) | 396 (0.1) | 0.01 | 0.01 |
| Liver disease | 220 (0.8) | 2807 (0.7) | 2458 (0.6) | 0.01 | 0.02 |
| Peptic ulcer | 705 (2.7) | 8904 (2.3) | 8536 (2.2) | 0.02 | 0.02 |
| Rheumatoid arthritis | 818 (3.1) | 11239 (3.0) | 9465 (2.4) | 0.01 | 0.04 |
| Blood pressure, mean (sd), mm Hg | | | | | |
| Systolic blood pressure | 137.4 (18.8) | 139.5 (19.3) | 139.6 (19.3) | 0.01 | 0.01 |
| Diastolic blood pressure | 82.1 (10.6) | 82.1 (10.5) | 82.1 (10.5) | 0.01 | 0.01 |
| Hospital admissions, mean (sd) † | 0.59 (2.81) | 0.36 (1.88) | 0.43 (2.12) | 0.09 | 0.06 |

Abbreviations: SD, standard deviation; MET, metabolic equivalent of task; ASMD, absolute standardized mean difference.

#Physical activity status was measured by the International Physical Activity Questionnaire (IPAQ).

†Data collected within past one year of $T_0$ from primary care records.

*ASMD ≤0.10 is considered good balance between comparison groups.

**Supplementary Table 3. Demographic and medical characteristics of the SARS-CoV-2 infection, contemporary control, and historical control groups(without history of mental health outcome two years before the start of follow-up) before weighting**

| Characteristics | SARS-CoV-2 infection (n=19353) | Contemporary control (n=301398) | Historical control (n=306,809) | ASMD between infection and contemporary control* | ASMD between infection and historical control* |
|---|---|---|---|---|---|
| Age, mean (sd) | 65.4 (8.4) | 68.6 (8.0) | 65.8 (8.0) | 0.39 | 0.05 |
| Sex, male (%) | 9518 (49.2) | 139728 (46.4) | 143922 (46.9) | 0.06 | 0.05 |
| Ethnicity, White (%) | 16136 (83.4) | 281946 (93.5) | 287253 (93.6) | 0.32 | 0.33 |
| Index of Multiple Deprivation, mean (sd) | 19.6 (14.2) | 16.7 (13.3) | 16.7 (13.3) | 0.21 | 0.21 |
| Body Mass Index, mean (sd) | 27.7 (4.7) | 27.0 (4.5) | 27.1 (4.5) | 0.15 | 0.14 |
| Current smoker (%) | 1822 (9.4) | 24835 (8.2) | 25528 (8.3) | 0.05 | 0.04 |
| Current drinker (%) | 17761 (91.8) | 279427 (92.7) | 284387 (92.7) | 0.04 | 0.04 |
| Physical activity, high level (%)[#] | 6561 (33.9) | 100922 (33.5) | 102511 (33.4) | 0.05 | 0.05 |
| Vaccination status, fully-vaccinated (%) | 7354 (38.0) | 116340 (38.6) | NA | 0.01 | NA |
| Medications (%)[†] | | | | | |
| Lipid lowering drugs | 5589 (28.9) | 98473 (32.7) | 85619 (27.9) | 0.08 | 0.02 |
| RAS inhibitors | 4008 (20.7) | 66194 (22.0) | 61584 (20.1) | 0.03 | 0.02 |
| Other anti-hypertensives | 1621 (8.4) | 27656 (9.2) | 27160 (8.9) | 0.03 | 0.02 |
| Anticoagulants | 605 (3.1) | 10996 (3.6) | 8135 (2.7) | 0.03 | 0.03 |
| Antiplatelet drugs | 1775 (9.2) | 29142 (9.7) | 28003 (9.1) | 0.02 | 0.01 |
| Proton pump inhibitors | 4883 (25.2) | 71385 (23.7) | 63799 (20.8) | 0.04 | 0.11 |
| Diabetes medicines | 1325 (6.8) | 17235 (5.7) | 15696 (5.1) | 0.05 | 0.07 |
| Systemic glucocorticoids | 908 (4.7) | 11640 (3.9) | 14236 (4.6) | 0.04 | 0.01 |
| Immunosuppressants | 195 (1.0) | 3085 (1.0) | 2843 (0.9) | 0.01 | 0.01 |
| Antineoplastic agents | 25 (0.1) | 280 (0.1) | 225 (0.1) | 0.01 | 0.02 |
| Coexisting conditions (%)[†] | | | | | |
| Acquired immunodeficiency syndrome | 22 (0.1) | 277 (0.1) | 257 (0.1) | 0.01 | 0.01 |
| Cancer | 1644 (8.5) | 31030 (10.3) | 26219 (8.5) | 0.07 | 0.01 |
| Cerebrovascular disease | 321 (1.7) | 5853 (1.9) | 4629 (1.5) | 0.02 | 0.01 |
| Chronic obstructive pulmonary disease | 3054 (15.8) | 44261 (14.7) | 41204 (13.4) | 0.03 | 0.07 |
| Chronic kidney disease | 832 (4.3) | 13944 (4.6) | 10920 (3.6) | 0.02 | 0.04 |

| | | | | | |
|---|---|---|---|---|---|
| Congestive heart failure | 243 (1.3) | 3567 (1.2) | 2294 (0.7) | 0.01 | 0.05 |
| Dementia | 160 (0.8) | 1557 (0.5) | 784 (0.3) | 0.04 | 0.08 |
| Diabetes (uncomplicated) | 1784 (9.2) | 24664 (8.2) | 21054 (6.9) | 0.04 | 0.09 |
| Diabetes (end-organ damage) | 568 (2.9) | 7672 (2.5) | 6286 (2.0) | 0.02 | 0.06 |
| Hemiplegia | 16 (0.1) | 218 (0.1) | 211 (0.1) | 0.01 | 0.01 |
| Liver disease | 121 (0.6) | 1850 (0.6) | 1635 (0.6) | 0.01 | 0.01 |
| Peptic ulcer | 425 (2.2) | 5993 (2.0) | 5548 (1.8) | 0.02 | 0.03 |
| Rheumatoid arthritis | 452 (2.3) | 7431 (2.5) | 6090 (2.0) | 0.01 | 0.02 |
| Blood pressure, mean (sd), mm Hg | | | | | |
| Systolic blood pressure | 137.2 (18.7) | 139.6 (19.3) | 139.8 (19.3) | 0.13 | 0.14 |
| Diastolic blood pressure | 82.1 (10.6) | 82.1 (10.5) | 82.2 (10.5) | 0.01 | 0.01 |
| Hospital admissions, mean (sd) † | 0.39 (2.3) | 0.27 (1.6) | 0.32 (1.8) | 0.02 | 0.03 |

Abbreviations: SD, standard deviation; MET, metabolic equivalent of task; ASMD, absolute standardized mean difference.

#Physical activity status was measured by the International Physical Activity Questionnaire (IPAQ).

†Data collected within past one year of $T_0$ from primary care records.

*ASMD ≤0.10 is considered good balance between comparison groups.

**Supplementary Table 4. Demographic and medical characteristics of the SARS-CoV-2 infection, contemporary control, and historical control groups (without history of mental health outcome two years before the start of follow-up) after weighting**

| Characteristics | SARS-CoV-2 infection (n=19353) | Contemporary control (n=301398) | Historical control (n=306,809) | ASMD between infection and contemporary control* | ASMD between infection and historical control* |
|---|---|---|---|---|---|
| Age, mean (sd) | 68.2 (8.3) | 68.4 (8.1) | 66.8 (8.1) | 0.02 | 0.06 |
| Sex, male (%) | 17805 (47.1) | 140150 (46.5) | 144200 (47.0) | 0.01 | 0.02 |
| Ethnicity, White (%) | 17805 (92.0) | 279999 (92.9) | 285332 (93.0) | 0.04 | 0.01 |
| Index of Multiple Deprivation, mean (sd) | 17.5 (13.2) | 16.9 (13.4) | 16.9 (13.4) | 0.05 | 0.01 |
| Body Mass Index, mean (sd) | 27.2 (4.3) | 27.1 (4.5) | 27.10 (4.5) | 0.02 | 0.01 |
| Current smoker (%) | 1684 (8.7) | 25016 (8.3) | 25772 (8.4) | 0.02 | 0.01 |
| Current drinker (%) | 17805 (92.0) | 279095 (92.6) | 284105 (92.6) | 0.03 | 0.01 |
| Physical activity, high level (%)[#] | 6425 (33.2) | 100968 (33.5) | 102474 (33.4) | 0.01 | 0.01 |
| Vaccination status, fully-vaccinated (%) | 7935 (41.0) | 116340 (38.6) | NA | 0.05 | NA |
| Medications (%)[†] | | | | | |
| Lipid lowering drugs | 6445 (33.3) | 97653 (32.4) | 100940 (32.9) | 0.02 | 0.03 |
| RAS inhibitors | 4335 (22.4) | 66006 (21.9) | 61669 (20.1) | 0.01 | 0.08 |
| Other anti-hypertensives | 1858 (9.6) | 27427 (9.1) | 26999 (8.8) | 0.02 | 0.05 |
| Anticoagulants | 735 (3.8) | 10850 (3.6) | 8284 (2.7) | 0.01 | 0.02 |
| Antiplatelet drugs | 1955 (10.1) | 28934 (9.6) | 27920 (9.1) | 0.01 | 0.07 |
| Proton pump inhibitors | 4741 (24.5) | 71733 (23.8) | 64430 (21.0) | 0.02 | 0.06 |
| Diabetes medicines | 1200 (6.2) | 17481 (5.8) | 15954 (5.2) | 0.02 | 0.02 |
| Systemic glucocorticoids | 793 (4.1) | 11755 (3.9) | 14113 (4.6) | 0.01 | 0.01 |
| Immunosuppressants | 232 (1.2) | 3014 (1.0) | 2761 (0.9) | 0.01 | 0.01 |
| Antineoplastic agents | 19 (0.1) | 301 (0.1) | 307 (0.1) | 0.01 | 0.01 |
| Coexisting conditions (%)[†] | | | | | |
| Acquired immunodeficiency syndrome | 20 (0.1) | 24 (0.1) | 25 (0.1) | 0.01 | 0.01 |
| Cancer | 1993 (10.3) | 30441 (10.1) | 31601 (10.3) | 0.01 | 0.01 |
| Cerebrovascular disease | 387 (2.0) | 5727 (1.9) | 5215 (1.7) | 0.01 | 0.02 |
| Chronic obstructive pulmonary disease | 2980 (15.4) | 44607 (14.8) | 44794 (14.6) | 0.02 | 0.02 |
| Chronic kidney disease | 948 (4.9) | 13864 (4.6) | 14113 (4.6) | 0.01 | 0.02 |

| | | | | | |
|---|---|---|---|---|---|
| Congestive heart failure | 252 (1.3) | 3617 (1.2) | 3374 (1.1) | 0.01 | 0.01 |
| Dementia | 116 (0.6) | 1507 (0.5) | 1227 (0.4) | 0.01 | 0.01 |
| Diabetes (uncomplicated) | 1722 (8.9) | 25016 (8.3) | 24545 (8.0) | 0.02 | 0.01 |
| Diabetes (end-organ damage) | 523 (2.7) | 7836 (2.6) | 7977 (2.6) | 0.01 | 0.01 |
| Hemiplegia | 15 (0.1) | 23 (0.1) | 22 (0.1) | 0.01 | 0.01 |
| Liver disease | 136 (0.7) | 1809 (0.6) | 1841 (0.6) | 0.01 | 0.01 |
| Peptic ulcer | 406 (2.1) | 6028 (2.0) | 5523 (1.8) | 0.01 | 0.02 |
| Rheumatoid arthritis | 464 (2.4) | 7535 (2.5) | 7057 (2.3) | 0.01 | 0.02 |
| Blood pressure, mean (sd), mm Hg | | | | | |
| Systolic blood pressure | 139.2 (19.4) | 139.4 (19.3) | 139.6 (19.3) | 0.01 | 0.01 |
| Diastolic blood pressure | 82.2 (10.5) | 82.1 (10.5) | 82.2 (10.5) | 0.01 | 0.01 |
| Hospital admissions, mean (sd) † | 0.31 (1.54) | 0.28 (1.89) | 0.33 (1.86) | 0.02 | 0.01 |

Abbreviations: SD, standard deviation; MET, metabolic equivalent of task; ASMD, absolute standardized mean difference.

#Physical activity status was measured by the International Physical Activity Questionnaire (IPAQ).

†Data collected within past one year of $T_0$ from primary care records.

*ASMD ≤0.10 is considered good balance between comparison groups.

**Supplementary Table 5. Number of individuals with a history of mental health outcome in the past two years before the start of follow up**

| Mental health outcome | Number† |
|---|---|
| **Psychiatric diagnoses**# | |
| Psychotic disorders | 484 |
| Mood disorders | 8254 |
| Mania/Bipolar affective disorder | 541 |
| Depressive episode | 7794 |
| Anxiety disorders | 7217 |
| Phobic anxiety disorders | 534 |
| Panic disorder | 514 |
| Generalized anxiety disorder | 6561 |
| Posttraumatic stress disorder | 177 |
| Substance use disorders | 6558 |
| Alcohol use disorder | 1795 |
| Tobacco use disorder | 5109 |
| Sleep disorders | 3225 |
| **Psychotropic medications**# | |
| Antipsychotics | 290 |
| Antidepressant | 25662 |
| SSRI | 22781 |
| SNRI | 3265 |
| Other antidepressant drugs | 9243 |
| Benzodiazepines | 13628 |
| Mood stabilizers | 16998 |
| Opioids | 32127 |
| **Composite outcomes**# | |
| Any psychiatric diagnosis | 20185 |
| Any prescriptions for psychotropic medications | 76351 |
| Any mental health related conditions | 85687 |

#There is overlap in numbers as participants may have history of multiple psychiatric diagnoses or psychotropic medications.
†Participants were excluded from the incident analyses (the infection group vs contemporary control).

**Supplementary Table 6. Risks of composite incident mental health outcomes after SARS-CoV-2 infection compared with the test-negative control group**

| Control group | Any psychiatric diagnosis (95% CI) | | Any psychotropic prescription (95%) | | Any mental health outcome (95% CI) | |
|---|---|---|---|---|---|---|
| | HR | ARD | HR | ARD | HR | ARD |
| Test-negative | 0.70 (0.64-0.76) | -15.29 (-18.36 to -11.93) | 0.97 (0.82-1.15) | -1.07 (-7.03 to 5.99) | 0.73 (0.57 -0.92) | -18.91 (-29.54 to -5.42) |

**Supplementary Table 7. Positive and negative outcome controls**

| Outcome [ICD-10 codes] | Hazard Ratio (95% CI) # |
|---|---|
| **Positive outcome controls** | |
| Fatigue [G93.3 and R53] | 2.76 (2.38-3.20) |
| Dyspnea [R06.0] | 2.98 (2.59-3.44) |
| **Negative outcome controls** | |
| Neoplasms of skin [C43-C44] | 0.86 (0.69-1.06) |
| Follicular cysts of skin [L72.0-L72.1] | 1.41 (0.91-2.20) |

#Risk of incident outcome between the SARS-CoV-2 infection group and contemporary control using the same analytic approach as in the main analyses of mental health outcomes; Outcomes of positive and negative controls were ascertained after the SARS-CoV-2 infection until the end of follow-up. Hazard ratios were adjusted for predefined and data-driven covariates. Participants with the history of the outcomes in the past two years before the start of follow up.

**Supplementary Table 8. Sensitivity analyses of the risks of composite incident mental health outcomes**

| Analysis | Any psychiatric diagnosis (95% CI) | Any psychotropic prescription (95%) | Any mental health related outcome (95% CI) |
|---|---|---|---|
| | HR | HR | HR |
| Main analyses (SARS-CoV-2 infection vs contemporary control) | | | |
| Using 1:10 PS matching | 1.39 (1.29-1.49) | 1.41 (1.30-1.53) | 1.29 (1.21-1.38) |
| Using data-driven covariates within three years | 1.30 (1.19-1.42) | 1.63 (1.49-1.79) | 1.48 (1.38-1.60) |
| Excluding participants with history of mental health outcomes in five years before follow-up | 1.30 (1.18-1.44) | 1.71 (1.53-1.90) | 1.64 (1.49-1.82) |
| Analyses stratified by test setting | | | |
| Hospitalized vs non-hospitalized COVID-19 patients | 1.40 (1.04-1.87) | 1.74 (1.24-2.43) | 1.65 (1.22-2.25) |

**Supplementary Table 9. Methodological pitfalls in long-COVID study using electronic health records compared with community-based cohort study**

| | EHR-based study (MVP,[1] TriNetX,[2] and QResearch[3]) | Community-based cohort study (UK Biobank[4]) | Limitations of EHR compared with population cohort on result interpretation[†] |
|---|---|---|---|
| Population selection | - Participants must be enrolled in the system and receive care within the structure the EHR represents | - Participants were randomly recruited at baseline and more representative of the general population | · Prone to sample selection bias<br>· Unspecific target population<br>· Findings had limited generalizable beyond that EHR population |
| Study design | - Retrospective (looking backward)<br>- Routinely collected data was retrospectively reviewed<br>- A relatively large sample size[‡] | - Prospective (looking forward)<br>- Participants were prospectively followed up for study outcomes<br>- A relatively modest to large sample size | · Outcome have already occurred in some of participants before study design<br>· Prone to recall bias or misclassification bias<br>· Causal interpretation of the findings is challenging<br>· Inferior level of evidence compared with prospective cohort studies |
| Definitions of variables | - Variables were defined after study design and extracted from data that were previously recorded for reasons not relating to the project. Some needed factors may not available in the records<br>- Variables may be difficult to measure correctly or have large inter-observer variability | - A series of variables were measured using standardized methods such as questionnaire, including detailed lifestyle, socioeconomic, medication use, and comorbidity factors | · Poor control over the exposure, covariates, and potential confounders<br>· Not all outcomes and exposures are formally adjudicated, as the database is dependent on coding by individual practitioners<br>·Inadequate adjustment of important confounders may result in residual confounding |
| Vulnerability to the effect of COVID-19 pandemic | - Due to the disruptions in patient care and reduced availability of services during the pandemic, the help-seeking behaviors of some individuals may be changed, such as avoiding hospital/outpatient care | - Participants were recruited and started to follow up before the pandemic | · Vulnerable to recording or detection bias in the setting of pandemic<br>·The effect of pandemic on help-seeking behaviors or access to health care may also impact cohort study if the outcomes are identified through data linkage to heath registry |

[†]Although some of these biases may also affect traditional population-based cohort, they are more common in research using EHR data. The potential for confounding is arguably larger in EHR data because the quality is often lower than that of traditional cohort. Note that the actual magnitude of unmeasured confounding likely depends on the comparison that is being made (e.g., COVID-19 vs non-infection/test-negative group here). In addition, confounding only plays a role in studies that aim to investigate causal effects.

[‡]Large sample sizes generally do not affect underlying biases, although they do however increase the precision of statistical tests, meaning that biased results are more likely to become statistically significant.

[1]**Million Veteran Program (US)**: Xie, Y., et al. Risks of mental health outcomes in people with covid-19: cohort study. *Bmj*.

[2]**TriNetX (US)**: Taquet, M., et al. 6-month neurological and psychiatric outcomes in 236 379 survivors of COVID-19: a retrospective cohort study using electronic health records. *Lancet Psychiatry*.

[3]**QResearch (UK)**: Clift, A. K. et al. Neuropsychiatric Ramifications of Severe COVID-19 and Other Severe Acute Respiratory Infections. *JAMA Psychiatry*.

[4]**UK Biobank (UK)**: Wang Y, et al. Long-term risk of psychiatric disorder and psychotropic prescription after SARS-CoV-2 infection among UK general population. *Nature Hum Behav*.
