# Supplementary material for: Long-term risk of psychiatric disorder and psychotropic prescription after SARS-CoV-2 infection among UK general population
Source: Nat Hum Behav. 2024 Mar 21;8(6):1076–87. doi: 10.1038/s41562-024-01853-4 (PMC11199144; doi:10.1038/s41562-024-01853-4)
Supplement: Supplementary file 2 — Reporting Summary [file 41562_2024_1853_MOESM2_ESM.pdf]

nature portfolio

Corresponding author(s): Junqing Xie

Last updated by author(s): Feb 18, 2024

Reporting Summary

Nature Portfolio wishes to improve the reproducibility of the work that we publish. This form provides structure for consistency and transparency in reporting. For further information on Nature Portfolio policies, see our Editorial Policies and the Editorial Policy Checklist.

Statistics

For all statistical analyses, confirm that the following items are present in the figure legend, table legend, main text, or Methods section.

n/a	Confirmed	
☐	☒	The exact sample size (*n*) for each experimental group/condition, given as a discrete number and unit of measurement
☒	☐	A statement on whether measurements were taken from distinct samples or whether the same sample was measured repeatedly
☐	☒	The statistical test(s) used AND whether they are one- or two-sided<br>*Only common tests should be described solely by name; describe more complex techniques in the Methods section.*
☐	☒	A description of all covariates tested
☐	☒	A description of any assumptions or corrections, such as tests of normality and adjustment for multiple comparisons
☐	☒	A full description of the statistical parameters including central tendency (e.g. means) or other basic estimates (e.g. regression coefficient) AND variation (e.g. standard deviation) or associated estimates of uncertainty (e.g. confidence intervals)
☐	☒	For null hypothesis testing, the test statistic (e.g. *F*, *t*, *r*) with confidence intervals, effect sizes, degrees of freedom and *P* value noted<br>*Give P values as exact values whenever suitable.*
☒	☐	For Bayesian analysis, information on the choice of priors and Markov chain Monte Carlo settings
☐	☒	For hierarchical and complex designs, identification of the appropriate level for tests and full reporting of outcomes
☐	☒	Estimates of effect sizes (e.g. Cohen's *d*, Pearson's *r*), indicating how they were calculated

Our web collection on statistics for biologists contains articles on many of the points above.

Software and code

Policy information about availability of computer code

Data collection	No software was used.
Data analysis	All analyses and data visualizations were conducted using R statistical software (version 4.1).

For manuscripts utilizing custom algorithms or software that are central to the research but not yet described in published literature, software must be made available to editors and reviewers. We strongly encourage code deposition in a community repository (e.g. GitHub). See the Nature Portfolio guidelines for submitting code & software for further information.

Data

Policy information about availability of data

All manuscripts must include a data availability statement. This statement should provide the following information, where applicable:

- Accession codes, unique identifiers, or web links for publicly available datasets
- A description of any restrictions on data availability
- For clinical datasets or third party data, please ensure that the statement adheres to our policy

Researchers can apply to use the UK Biobank dataset by registering and applying at https://ukbiobank.ac.uk/register-apply/. Any additional summary data generated and/or analyzed during the current study are available from the corresponding author on reasonable request.

Human research participants

Policy information about studies involving human research participants and Sex and Gender in Research.

Reporting on sex and gender	This large prospective cohort of 406,579 adults included 224,681 women and 181,898 men, with a mean [SD] age of 66.1 [8.4] years. All participants provided informed written consent to take part in the study and be followed-up through linkage to health-related records. The main analyses were assessed in subgroups based on sex and other population characteristics such as age, ethnicity, and BMI.
Population characteristics	In this prospective cohort of 406,579 adults (224,681 women, 181,898 men; mean [SD] age 66.1 [8.4] years), 26,181 had a positive test for SARS-CoV-2. The primary comparison cohorts comprised 26,181 participants in the SARS-COV-2 infection group, 380,398 in the contemporary control group and 384,030 in the historical control group. Detailed demographic and medical characteristics of all comparison cohorts before and after weighting were shown. For example, before weighting, participants in the infection group were younger (mean age: 66.0 years vs 68.8 years), less likely from the White ethnic group (84.6% vs 93.7%), more socioeconomically deprived (mean IMD: 20.5 vs 17.3), and more physically obese (mean BMI: 28.1 vs 27.3), compared with contemporary controls. After weighting, all covariates are balanced (ASMD<0.1).
Recruitment	The UK Biobank is an ongoing community-based prospective cohort study, which recruited more than 500,000 participants out of 9.2 million adults aged 40-69 years in the UK who were identified from National Health Service and invited to participants (5.5% response rate). The baseline survey took place from 2006 to 2010 in 22 assessment centers.
Ethics oversight	This study was based on data from UK Biobank. All participants provided written informed consent at the UK Biobank cohort recruitment. This study received ethical approval from UK Biobank Ethics Advisory Committee (EAC) and was performed under the application of 65397.

Note that full information on the approval of the study protocol must also be provided in the manuscript.

Field-specific reporting

Please select the one below that is the best fit for your research. If you are not sure, read the appropriate sections before making your selection.

☒ Life sciences ☐ Behavioural & social sciences ☐ Ecological, evolutionary & environmental sciences

For a reference copy of the document with all sections, see nature.com/documents/nr-reporting-summary-flat.pdf

Life sciences study design

All studies must disclose on these points even when the disclosure is negative.

Sample size	We included UK Biobank participants from England who were still alive by March 1, 2020 (N=406,579) to construct infected and control cohorts. We further excluded those with the history of mental health outcomes one year before the start of follow-up (327,022 participants in the contemporary control, 21,307 in the COVID-19 group, and 332,740 in the historical control). To our knowledge, the UK Biobank including about half a million participants is one of the current largest random well-controlled population-based cohort with detailed and robust recording of confounding factors that were largely unavailable in previous studies based on electronic health records. The large sample size obtained were deemed to provide reliable risk estimates of mental health outcomes.
Data exclusions	Participants with the history of mental health outcomes in one or two year before the start of follow-up were excluded to avoid potential reverse causality.
Replication	The main aim of the current study is to assess the risk of mental health outcomes after COVID-19 compared to uninfected control. In the main analyses, we used individuals with no evidence of SARS-CoV-2 infection but exposed similar pandemic-related environmental stressors as contemporary control group. For replication of major analyses, in the same study, we constructed a historical control cohort predating the pandemic and then compared the risk of mental health outcomes following SARS-CoV-2 infection compared with those unaffected by the COVID-19 pandemic. The results using the historical control cohort were consistent with the main analyses of contemporary control.
Randomization	No randomization was required as all samples were included in the analysis
Blinding	No blinding was applicable to this observational study as no intervention were applied to participants.

Reporting for specific materials, systems and methods

We require information from authors about some types of materials, experimental systems and methods used in many studies. Here, indicate whether each material, system or method listed is relevant to your study. If you are not sure if a list item applies to your research, read the appropriate section before selecting a response.

Materials & experimental systems

n/a	Involved in the study	
☒	☐	Antibodies
☒	☐	Eukaryotic cell lines
☒	☐	Palaeontology and archaeology
☒	☐	Animals and other organisms
☒	☐	Clinical data
☒	☐	Dual use research of concern

Methods

n/a	Involved in the study	
☒	☐	ChIP-seq
☒	☐	Flow cytometry
☒	☐	MRI-based neuroimaging
